# Introducing the University of California, Irvine Corneal Cystine Crystal Score: A Novel Tool for Assessing Corneal Crystal Deposition in Cystinosis Patients

**DOI:** 10.1038/s41598-025-20108-4

**Published:** 2025-10-16

**Authors:** Kimia Rezaei, Kourosh Shahraki, Lauren Chen, Shaden H. Yassin, Olivia L. Lee, Kianoush Shahraki, Donny W. Suh

**Affiliations:** 1 University of California Riverside, School of Medicine, Riverside, CA USA; 2 Gavin Herbert Eye Institute (GHEI), University of California, Irvine, CA USA; 3 Shiley Eye Institute, University of California San Diego, San Diego, CA USA; 4 Farabi Eye Hospital, Tehran University of Medical Sciences, Tehran, Iran

**Keywords:** Cystinosis, Corneal crystal deposition, UCI cystinosis corneal crystal scoring (CCCS), Corneal grading system, Corneal diseases, Paediatric research, Experimental models of disease

## Abstract

This study aimed to develop and validate the University of California Irvine Corneal Cystine Crystal Score (UCI CCCS), a novel grading system for objectively assessing corneal crystal deposition in cystinosis patients using a slit lamp biomicroscope. This tool provides a standardized method for monitoring disease progression and evaluating treatment efficacy. This retrospective study analyzed clinical records from 36 cystinosis patients examined at the Day of Hope conference, all with the infantile nephropathic form. The records included evaluations from slit-lamp examinations and Best Corrected Visual Acuity (BCVA). After evaluation, the UCI CCCS was developed by experts in pediatric ophthalmology and cornea specialty; this system differentiates cystine crystal density, iris visibility, and corneal haze on a scale from 0 to 5. For validation, 107 slit-lamp images were assessed by masked graders. Statistical analysis using Intraclass Correlation Coefficient (ICC) and Cohen’s kappa was performed to confirm the tool’s ability to produce consistent, reproducible results across and within graders. The cohort included 23 males and 13 females (mean age: 15.64 years, range: 0–60). A total of 72 eyes were evaluated. In the validation phase, the UCI CCCS demonstrated high inter-rater agreement (κ = 0.869, P < 0.001) and excellent intra-rater reliability (ICC = 0.922, P < 0.001). The UCI CCCS is a validated, reliable, and reproducible tool for assessing corneal crystal involvement in cystinosis, supporting its clinical and research applications.

**Supplementary Information:**

The online version contains supplementary material available at 10.1038/s41598-025-20108-4.

## Introduction

Cystinosis is a rare autosomal recessive lysosomal storage disorder affecting about 1 in every 100,000 to 200,000 live births^1^. The disease is caused by mutations in the CTNS gene, which encodes the cystinosin protein responsible for transporting cystine out of the lysosome^2,3^. Defective cystinosin protein accumulates cystine crystals in lysosomes throughout the body, including the eye, resulting in multi-organ dysfunction^2^.

Cystinosis is clinically classified into three major subtypes based on age of onset and systemic involvement: infantile nephropathic cystinosis, the most common and severe form, characterized by early-onset renal failure and multisystem involvement; juvenile (intermediate) cystinosis, which presents later in childhood with milder and slower systemic progression; and ocular (benign) cystinosis, which primarily involves the cornea without significant renal or systemic disease^1–3^. This clinical heterogeneity is important to consider in both diagnosis and monitoring.

Corneal crystals typically start in infancy at the perilimbal region of the cornea and spread centrally and posteriorly over time^4^. They have a distinctive structure, appearing as fine, evenly shaped needles scattered throughout the corneal tissue^2^. These crystals build up in various parts of the eye, including the cornea itself, conjunctiva, iris, ciliary body, choroid, retinal pigment epithelium, and lens capsule^5^. One of the major clinical manifestations of cystinosis is corneal crystalline keratopathy, where cystine crystals deposit within the cornea, leading to photophobia, blepharospasm, and potential vision loss if untreated^3,6^. Moreover, corneal crystal accumulation can lead to recurrent corneal erosions, filamentary keratopathy, band-shaped keratopathy, severe peripheral corneal neovascularization, and scarring^7,8^.

Several grading systems have been suggested to evaluate the extent of corneal crystal deposition, but none universally correlates effectively with clinical symptoms and findings^9–12^. The corneal cystine crystal score (CCCS) was developed by Gahl et al. at the NIH and grades corneal severity on a scale of 0.00 to 3.00 at 0.25 increments^9^. However, this scale is clinically difficult to distinguish between 0.25 incremental differences in severity, and this score does not describe the full spectrum of corneal disease severity. The retinochoroidal cystine crystal score is based on spectral domain optical coherence tomography (SD-OCT) imaging to characterize chorioretinal cystine crystals^6,9,10^. However, a uniform system that accurately captures the clinical presentation and full progression of the disease in the cornea has yet to be established. The limited range of existing grading systems complicates objective monitoring of ocular cystinosis progression and treatment efficacy, particularly as patients’ increased longevity with modern therapies may lead to disease severity exceeding these scales’ upper bounds. To address this knowledge gap, we developed a new scoring system to objectively grade the extent and distribution of corneal crystal deposition based on the slit-lamp biomicroscope. The University of California, Irvine Corneal Cystine Crystal Score (UCI CCCS) is a quantitative score that ranges from 0 to 5 and aims to provide a standardized method to monitor the disease progression of cystinosis corneal keratopathy.

## Materials and methods

This retrospective study utilized the data collected at the Day of Hope Conference, organized by the Cystinosis Research Foundation, and approved by the Institutional Review Board (IRB) of the University of California, Irvine (IRB #5108). Under institutional guidelines, the study was designated as Non-Human Research (NHR), as all ophthalmologic evaluations were performed as part of routine clinical care rather than for research purposes. Due to its retrospective nature, requirement for informed consent was waived by the Institutional Review Board of the University of California, Irvine. The study protocol was reviewed and approved by the UCI IRB, ensuring compliance with institutional and ethical research guidelines. All procedures adhered to the Declaration of Helsinki and its amendments.

### Patient population and clinical data

This study was designed as a retrospective study utilizing clinical records from thirty-six patients with confirmed diagnoses of cystinosis who were examined at the annual Day of Hope conference by the Cystinosis Research Foundation. All patients had the infantile nephropathic form, the most common and severe phenotype of the disease. Diagnosis of cystinosis was confirmed through a combination of previous genetic testing, clinical history, and documented systemic cysteamine therapy use.

Demographic variables included in the dataset were patient age, gender, and duration of cystinosis diagnosis. Additionally, records contained information regarding ophthalmic medication history, including the use of cysteamine eye drops, duration of therapy, and adherence to prescribed treatment regimens.

Ophthalmologic examinations were conducted as part of routine clinical care by an expert ophthalmologist (D.W.S.) specializing in pediatric ophthalmology. Clinical records included best-corrected visual acuity (BCVA) measurements and detailed slit-lamp examination (SLE) findings.

### Grading system development

**Fig. 1:**
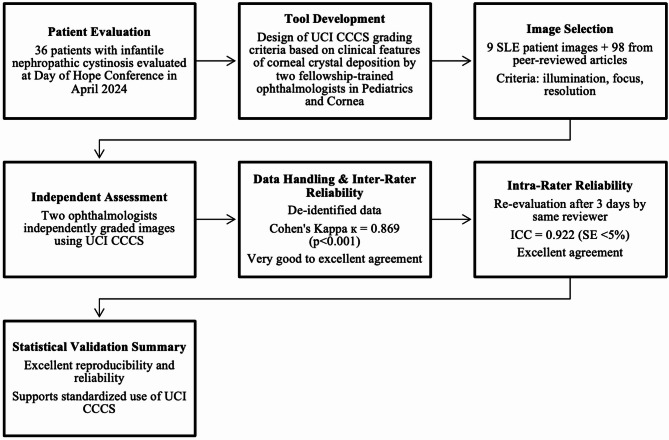
Workflow for the development and validation of the UCI Corneal Cystine Crystal Score (UCI CCCS), detailing grading criteria design, image selection, independent ophthalmologist assessment, and reliability testing with Cohen’s Kappa and Intraclass Correlation Coefficient (ICC).

**Fig. 2:**
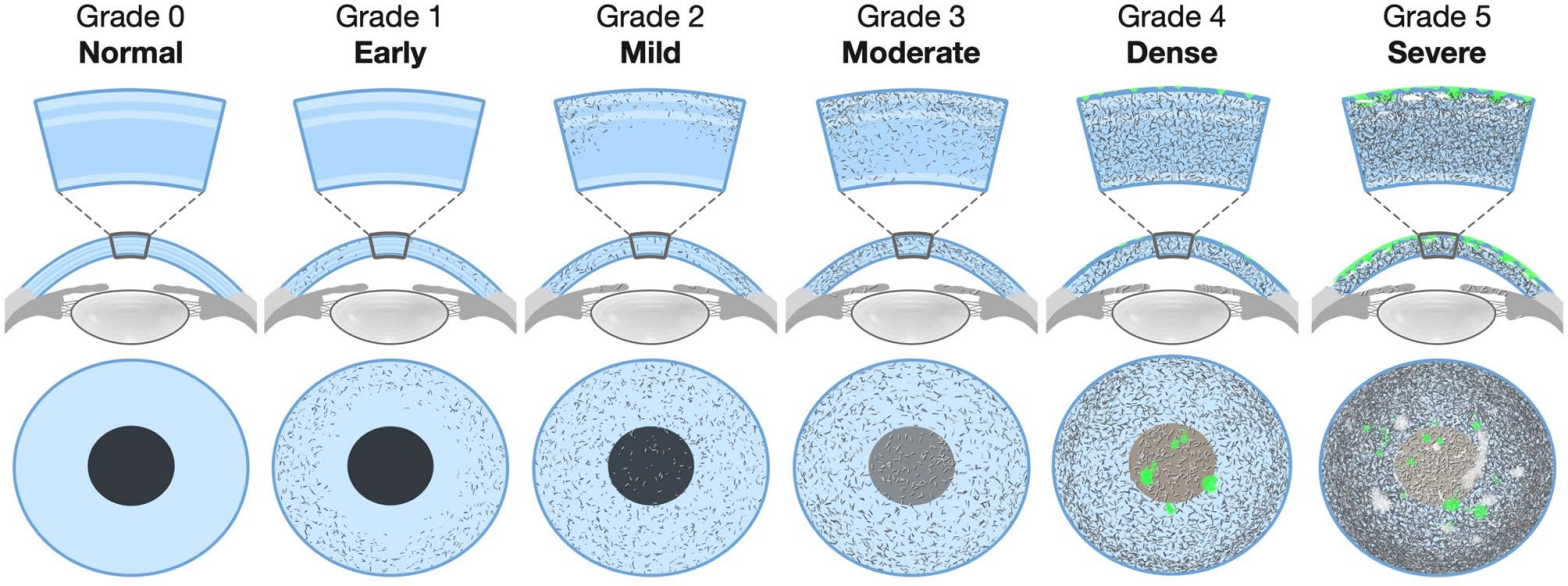
University of California, Irvine Corneal Cystine Crystal Score (UCI-CCCS) System**.** Schematic illustrations of grades 0–5 demonstrating the progressive accumulation of corneal cystine crystals, reduction in iris visibility, and increasing severity from normal (Grade 0) to severe (Grade 5).

The grading system was developed and validated through a multi-step process, including patient evaluation, image selection, independent grading, and statistical reliability testing (Fig. 1). Initially, the clinical findings from the examination of 36 cystinosis patients at the Day of Hope Conference served as the foundation for the development of the UCI CCCS grading system. Drawing from these real-world observations, two experts in pediatric ophthalmology and Corneal diseases, D.W.S. and O.L.L., developed the UCI CCCS grading system based on the evaluation of patients seen at the Day of Hope conference. Through consensus discussions, extensive experience in providing a standard of care for cystinosis patients, and a comprehensive review of existing literature on corneal manifestations associated with cystinosis, the grading criteria were developed to ensure clear differentiation between the severity of cystine crystal deposition and its effects on corneal transparency and iris visibility. The grading system is based on scores ranging from 0 to 5, reflecting the cystine crystal density, iris visibility, and associated clinical features. Representative schematic illustrations for each grade are shown in Fig. 2, depicting the progressive increase in crystal density and reduction in iris visibility. A detailed description of the diagnostic criteria and clinical implications for each grade is provided in Table 1, outlining the specific features that distinguish each severity level.

**Table 1:** Detailed criteria for the university of California, Irvine corneal cystine crystal score (UCI-CCCS) grading system**.** This table summarizes the specific diagnostic features and clinical implications of each grade (0–5) in the UCI-CCCS system. Parameters include crystal distribution, Iris visibility as a surrogate for corneal haze, slit-lamp appearance, and additional findings such as punctate epithelial erosions, scars, or filaments at higher grades.

Grade	Crystal Distribution	Iris Visibility	Slit-lamp Appearance	Additional Features/Clinical Implication
**0**	No crystals present.	Iris details fully visible; cornea completely clear.	None.	Normal cornea.
**1**	Crystals confined to the peripheral cornea, sparing the central 3 mm.	Iris details fully visible, no distortion.	Peripheral crystalline deposits without stromal haze.	No functional visual impact.
**2**	Moderate accumulation of crystals involving both the peripheral and central cornea.	Iris and pupil margins are sharply defined, with no distortion.	Discrete, well-defined crystals with minimal surrounding haze; haze limited to the superficial one-third of the stroma.	Minimal effect on visual quality; typically, subclinical.
**3**	Increased crystal density, often clustered or confluent, in both peripheral and central cornea.	Iris and pupil details remain visible but appear blurred or softened.	Diffuse stromal haze extending into mid/posterior stroma; noticeable light scatter.	Mild reduction in contrast sensitivity or early visual disturbance, even if visual acuity remains intact.
**4**	Dense concentration of crystals across the cornea.	Iris details partially obscured, difficult to discern.	Full-thickness stromal haze with patchy or uniform opacity.	Presence of punctate epithelial erosions (PEE); definite visual impairment due to obscuration of fine iris detail.
**5**	Severe, confluent crystals in the central and peripheral cornea.	Iris completely obscured, not discernible.	Dense opacification; cornea diffusely opaque.	PEE, corneal epithelial or stromal scars, and filaments; severe visual limitation with profound reduction in visual quality.

The criteria for each grade were determined through careful deliberation, and the final grading definitions were established to provide objective and reproducible measures of corneal involvement in cystinosis. Scores were assigned based on the following factors:

As the UCI CCCS grading system was developed following the clinical evaluation of patients at the Day of Hope Conference, and because slit-lamp imaging was not consistently performed for all participants during the event, a comprehensive literature-based image dataset was assembled to facilitate validation. A total of 107 slit-lamp photographs were utilized, comprising nine images obtained from study participants and 98 high-quality images sourced from open-access peer-reviewed publications available on PubMed (Fig. S1). Images were selected based on predefined inclusion criteria, including adequate illumination, sharp focus, and sufficient resolution to ensure clear visualization of corneal features relevant to the CCCS criteria.

Two board-certified ophthalmologists, who were not involved in the grading system’s development, evaluated all images independently and in a masked fashion to reduce observer bias. To assess intra-rater reliability, one grader re-evaluated the complete image set after a three-day interval under identical masked conditions. The grading system was developed to provide a standardized, reproducible method for assessing corneal involvement in cystinosis, ensuring that both graders applied the same criteria when assigning scores.

### Data analysis

All patient data used in this study were de-identified before analysis. Statistical analyses were conducted to assess the intra- and inter-rater reliability of the UCI CCCS grading system. Agreement between graders was evaluated using the absolute agreement model of the intraclass correlation coefficient (ICC), where values between 0.81 and 1.00 were considered to indicate near-perfect agreement, 0.61–0.80 represented good agreement, and values below 0.40 indicated poor agreement. Additionally, inter-rater agreement was assessed using Cohen’s kappa (κ) statistic. A p-value of < 0.001 was considered statistically significant. All statistical analyses were performed using IBM SPSS Statistics for Mac, version 29.0.2.0 (IBM Corp., 2023).

## Results

This retrospective study included a cohort of 36 patients with various types of cystinosis, all of whom underwent comprehensive ophthalmological examinations at the Cystinosis Research Foundation’s annual Day of Hope Conference in April 2024. The cohort consisted of 23 males and 13 females, with a mean age of 15.64 years (range: 0 to 60 years) (Table 2). A total of 72 eyes were graded using a slit-lamp examination (SLE). In addition, anterior segment and fundus photographs of the eyes were independently graded after examination by the same expert graders.

**Table 2:** Patient demographics. Demographic data and best visual acuity measurements were extracted from medical records (recorded age corresponds to the age on the day of exam at the day of hope conference).

Total *N* = 36	
Gender (M: F, %Male)	23:13, 63.8%
Mean Age	15.64 ± 12.5
BCVA mean Snellen (*n* = 32)	20/23
mean logMAR (*n* = 32)	0.069
**Age** 0–4 5–10 11–20 21–30 31–40 41–60	**N** 6 8 15 2 3 2

A total of one hundred and seven slit-lamp photographs, including nine photographs from five participants and 98 images selected from peer-reviewed articles available on PubMed, were graded using the UCI CCCS system (Fig. S1). The publicly sourced images came from an unknown number of eyes, and each image was selected based on specific criteria, including appropriate illumination, fine focus, and high resolution, to effectively display corneal structures. The measurement system’s reliability was assessed using intra-rater and inter-rater reliability analyses, demonstrating strong consistency and reproducibility. Intra-rater reliability analysis revealed excellent agreement, with a kappa statistic of 0.922 (*p* < 0.001) and an intraclass correlation coefficient (ICC) of 0.922 (95% CI: 0.883–0.962), indicating almost perfect agreement. Inter-rater reliability analysis showed very good to excellent agreement, with a kappa of 0.869 (*p* < 0.001) and an ICC of 0.869 (95% CI: 0.830–0.909). Both reliability estimates showed high precision, with less than 5% standard error of the UCI CCCS system, which demonstrated robust inter-rater agreement (κ = 0.869) and excellent intra-rater reliability (ICC = 0.922), providing a reliable quantitative assessment of corneal crystal deposition across different stages of the disease (Table 3). These results suggest that the UCI CCCS system can effectively capture corneal involvement severity and monitor changes over time, supporting its use in research or clinical settings.

**Table 3:** Intra- and inter-grader agreement analysis on UCI cystinosis corneal crystal scoring (CCCS) system. The table presents kappa (κ) values, p-values, intraclass correlation coefficient (ICC) with 95% confidence intervals, and standard errors for intra-rater and inter-rater reliability. The UCI CCCS system demonstrated excellent reliability, with κ values of 0.922 for intra-rater and 0.869 for inter-rater reliability, with p-values < 0.001 and standard errors below 5%.

	Kappa	*P* value	ICC (95% interval)	Standard Error (%)
Intra-rater Reliability	0.922	< 0.001	0.883–0.962	< 5%
Inter-rater Reliability	0.869	< 0.001	0.830–0.909	< 5%

## Discussion

In this study, we introduce the UCI CCCS, a novel grading system for assessing corneal crystal deposition in patients with cystinosis. The UCI CCCS was designed to provide a reliable and reproducible method for grading the severity of corneal involvement, using a six-point scale to capture the full spectrum of corneal cystine crystal deposition. Our results demonstrate that this grading system has excellent intra-rater and very good intra-rater reliability, with high κ and ICC values, confirming its robustness and utility for both clinical practice and research purposes. The UCI CCCS offers a quantitative approach that can support long-term monitoring of disease progression and treatment efficacy in cystinosis patients, where corneal crystal deposition can have significant impacts on visual function.

Our review of the literature revealed that previous grading systems for ocular cystinosis have relied on various markers of disease progression, primarily focusing on crystal density within the cornea^9,11,12^. The Gahl scale, a commonly used corneal crystal scoring system, grades corneal crystal severity on a 0 to 3 scale at 0.25 increments. However, it was not successful in correlating with clinical symptoms, particularly in cases with subtle changes in crystal density^9^. Furthermore, the in vivo confocal microscopy (IVCM) scoring system, proposed by Labbé et al. in 2009, addresses the limitations of the CCCS by providing a more accurate evaluation of crystal density in different corneal layers^11^. While the IVCM scoring system has been recognized as a valuable tool, particularly in research settings and for monitoring treatment responses, its widespread use in routine clinical practice may be limited by the need for specialized equipment and expertise. Additionally, as many cystinosis patients are predominantly from the infant to toddler age group, positioning for IVCM in this age group is often difficult to achieve. Similarly, systems like the retinochoroidal cystine crystal grading based on SD-OCT imaging fail to capture the full range of corneal manifestations^10^.

Furthermore, validated imaging modalities such as Scheimpflug-based corneal densitometry and anterior segment optical coherence tomography (AS-OCT) have been increasingly utilized to quantify corneal cystine crystal deposition with high precision^13^. This allows for the detection of subtle changes in crystal density over time, which can be critical for evaluating disease progression and treatment response^13–15^. However, their implementation is often limited by cost, need for specialized equipment, and operator expertise, making them less accessible in many routine clinical environments or in resource-limited settings. In contrast, the UCI CCCS was intentionally designed as a slit-lamp–based grading system that leverages standard ophthalmic examination equipment, enabling rapid, reproducible, and clinically meaningful assessments without reliance on advanced imaging technologies. This approach allows for broader applicability in everyday practice, facilitates adoption in diverse care settings, and ensures that patients can still receive standardized evaluations even in the absence of high-cost imaging devices.

Recently, Liang et al. introduced the 3 C classification system, which categorizes patients based on crystal deposition, complications, and compliance with therapy^12^. While the 3 C system provides a more comprehensive assessment of disease severity, it still differs from the UCI CCCS in its complexity and inclusion of patient compliance. Our UCI CCCS offers a streamlined, cornea-focused approach that concentrates specifically on the extent of corneal crystal deposition and the impact on visual clarity without factoring in compliance or extra-corneal complications.

The UCI CCCS grading system is unique in several aspects. To our knowledge, this is the first assessment that integrates qualitative and quantitative measures, evaluating the density of corneal crystals and their effects on corneal clarity, haze, and iris visibility. This holistic approach allows for a more comprehensive evaluation compared to other methods that rely solely on crystal density. Additionally, the system’s ease of use makes it practical for clinical settings, where accurate yet straightforward grading is essential for routine monitoring. The six-point scale provides distinct and clinically meaningful cut-offs, ranging from no visible crystals (Grade 0) to severe crystal accumulation with significant corneal opacity and associated complications (Grade 5). These features make the UCI CCCS an efficient tool for capturing dynamic changes in corneal crystal deposition, particularly as patients respond to treatments such as cysteamine eye drops. The excellent reliability metrics also reinforce its potential for use in longitudinal studies, where consistent grading over time is critical.

The UCI CCCS system was applied to a diverse cohort of patients ranging widely in age, which allowed us to assess corneal crystal deposition across various stages of disease progression. Additionally, the images selected from published literature collected from PubMed search exhibited a broad range of crystal accumulation, corneal haze, and iris visibility, contributing to the comprehensive evaluation of cystinosis manifestations in this study. The subjects included in the present cohort demonstrated a wide spectrum of ocular involvement, from early crystal deposition to advanced corneal disease. All image measurements were conducted according to the standardized grading protocol described in this study, ensuring that our findings are representative and can be reasonably generalized to daily clinical practice for grading cystinosis.

This study, nevertheless, has several limitations. The UCI CCCS grading system was developed and validated using SLE images, representing the current gold standard for clinical assessment of corneal crystal deposition in cystinosis. At present, SLE is the only recommended and validated modality for applying the UCI CCCS. Other anterior segment imaging systems, such as high-resolution anterior segment cameras or Scheimpflug imaging (e.g., Pentacam), were not evaluated in this study and may not provide comparable crystal distribution or corneal haze visualization.

Furthermore, while our results demonstrate excellent inter- and intra-rater reliability, indicating strong reproducibility of the UCI CCCS, this alone does not establish its clinical utility. A key limitation of this study is the absence of direct comparison with established imaging-based modalities such as AS-OCT, IVCM, and Scheimpflug-based corneal densitometry. These techniques have been validated as reliable tools for quantifying corneal cystine crystal burden and monitoring disease progression in cystinosis, and thus represent important benchmarks for external validation. Future studies should focus on correlating UCI CCCS grades with these imaging modalities, as well as longitudinal clinical outcomes, to evaluate the tool’s predictive validity and its responsiveness to treatment. Such comparative analyses will be essential for establishing the clinical relevance of the UCI CCCS and supporting its broader adoption in both research and clinical care settings. The authors also acknowledged that future prospective studies are needed to evaluate the clinical applicability of the UCI CCCS through live grading by two independent examiners during slit-lamp examinations. Further studies are needed to assess whether these imaging modalities can be standardized and reliably used in conjunction with the UCI CCCS to expand its clinical utility and applicability.

An important limitation of this study is the variability in imaging quality and parameters among the slit-lamp photographs sourced from published literature. While these images provided a valuable resource for validation, differences in slit-beam width, magnification, illumination, and depth of focus may have influenced the consistency of grading across images. To mitigate this, we applied strict inclusion criteria, requiring high-resolution, well-focused, and appropriately illuminated images, to ensure that key corneal features were sufficiently visible for reliable grading. Each image was evaluated independently and scored in isolation, and our reliability analyses reflect agreement based on the specific characteristics of each image rather than cross-image comparisons. Nevertheless, future research should incorporate standardized imaging protocols to enhance reproducibility and enable meaningful longitudinal and inter-study comparisons, ideally with consistent slit-lamp settings and photographic techniques during image acquisition. Establishing such protocols will be essential for optimizing the clinical utility of the UCI CCCS in both real-world and research settings.

## Supplementary Information

Below is the link to the electronic supplementary material.

Supplementary Material 1

## Data Availability

The datasets used and analyzed during the development of the UCI CCCS system from the Day of Hope Conference, where patients with Cystinosis were evaluated and examined, are available from the corresponding author on reasonable request. The Slit-Lamp images gathered from Open Access Articles from a PubMed search are included in this published article in Supplementary Figure 1.
