# Supplementary material for: Introducing the University of California, Irvine Corneal Cystine Crystal Score: A Novel Tool for Assessing Corneal Crystal Deposition in Cystinosis Patients
Source: Sci Rep. 2025 Oct 16;15:36262. doi: 10.1038/s41598-025-20108-4 (PMC12533177; doi:10.1038/s41598-025-20108-4)
Supplement: Supplementary file 1 — Supplementary Material 1 [file 41598_2025_20108_MOESM1_ESM.docx]

| **Citation** | **SLE Image** |
| --- | --- |
| SLE image from participants | 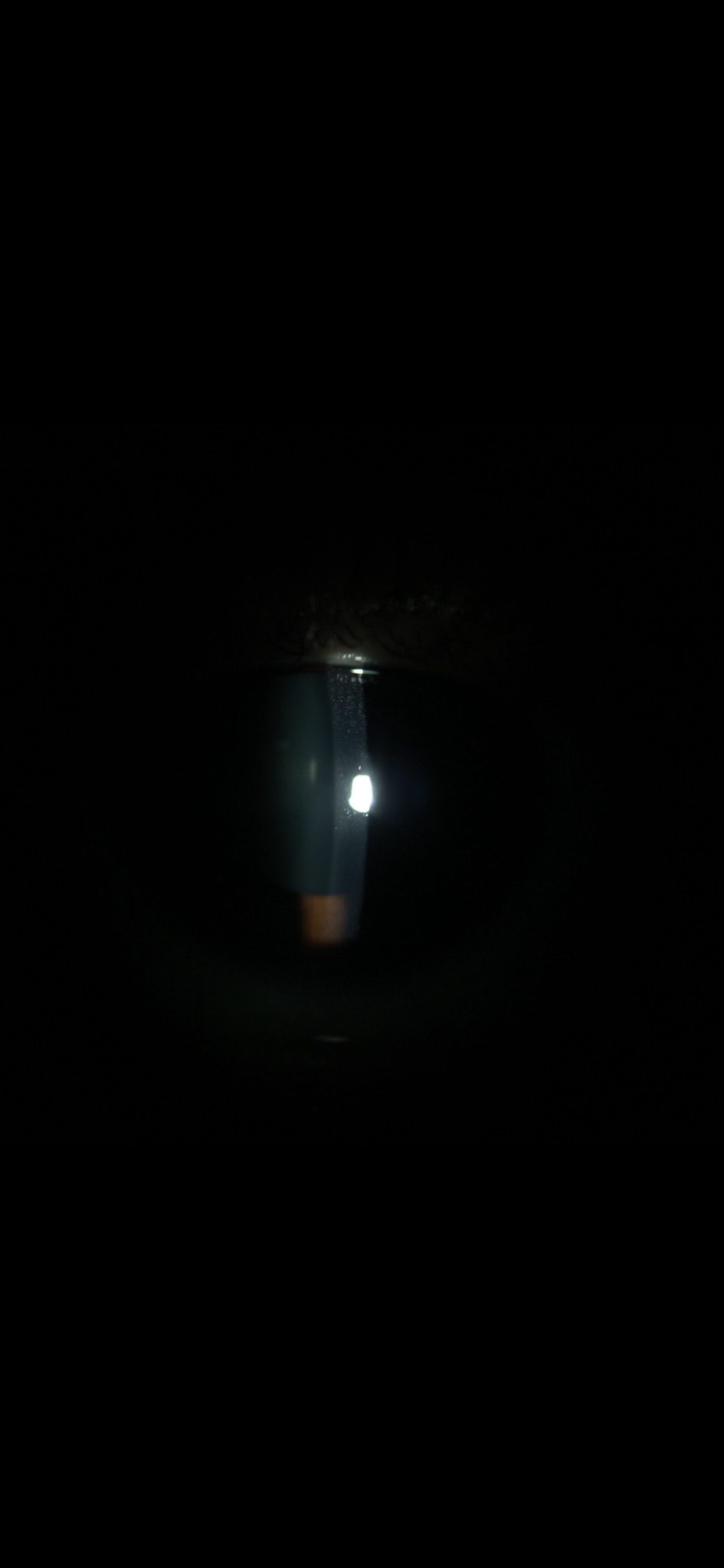 |
| Gahl WA, Reed GF, Thoene JG, et al. Cysteamine therapy for children with nephropathic cystinosis. *N Engl J Med*. 1987;316(16):971-977. doi:10.1056/NEJM198704163161602 | Representative Image is accessible via reference |
| Labbé A, Niaudet P, Loirat C, Charbit M, Guest G, Baudouin C. In vivo confocal microscopy and anterior segment optical coherence tomography analysis of the cornea in nephropathic cystinosis. *Ophthalmology*. 2009;116(5):870-876. doi:10.1016/j.ophtha.2008.11.021 | Representative Image is accessible via reference |
| Gahl WA, Reed GF, Thoene JG, et al. Cysteamine therapy for children with nephropathic cystinosis. *N Engl J Med*. 1987;316(16):971-977. doi:10.1056/NEJM198704163161602 | Representative Image is accessible via reference |
| SLE image from participants | Representative Image is accessible via reference |
| Gahl WA, Reed GF, Thoene JG, et al. Cysteamine therapy for children with nephropathic cystinosis. *N Engl J Med*. 1987;316(16):971-977. doi:10.1056/NEJM198704163161602 | Representative Image is accessible via reference |
| Gahl WA, Reed GF, Thoene JG, et al. Cysteamine therapy for children with nephropathic cystinosis. *N Engl J Med*. 1987;316(16):971-977. doi:10.1056/NEJM198704163161602 | Representative Image is accessible via reference |
| Kowalczyk M, Toro MD, Rejdak R, Załuska W, Gagliano C, Sikora P. Ophthalmic Evaluation of Diagnosed Cases of Eye Cystinosis: A Tertiary Care Center’s Experience. *Diagnostics*. 2020; 10(11):911. https://doi.org/10.3390/diagnostics10110911 | Representative Image is accessible via reference |
| Gahl WA, Reed GF, Thoene JG, et al. Cysteamine therapy for children with nephropathic cystinosis. *N Engl J Med*. 1987;316(16):971-977. doi:10.1056/NEJM198704163161602 | Representative Image is accessible via reference |
| 098Gahl WA, Reed GF, Thoene JG, et al. Cysteamine therapy for children with nephropathic cystinosis. *N Engl J Med*. 1987;316(16):971-977. doi:10.1056/NEJM198704163161602 | Representative Image is accessible via reference |
| Labbé A, Niaudet P, Loirat C, Charbit M, Guest G, Baudouin C. In vivo confocal microscopy and anterior segment optical coherence tomography analysis of the cornea in nephropathic cystinosis. *Ophthalmology*. 2009;116(5):870-876. doi:10.1016/j.ophtha.2008.11.021 | Representative Image is accessible via reference |
| Gahl WA, Reed GF, Thoene JG, et al. Cysteamine therapy for children with nephropathic cystinosis. *N Engl J Med*. 1987;316(16):971-977. doi:10.1056/NEJM198704163161602 | Representative Image is accessible via reference |
| Gahl WA, Reed GF, Thoene JG, et al. Cysteamine therapy for children with nephropathic cystinosis. *N Engl J Med*. 1987;316(16):971-977. doi:10.1056/NEJM198704163161602 | Representative Image is accessible via reference |
| Gahl WA, Reed GF, Thoene JG, et al. Cysteamine therapy for children with nephropathic cystinosis. *N Engl J Med*. 1987;316(16):971-977. doi:10.1056/NEJM198704163161602 | Representative Image is accessible via reference |
| Gahl WA, Reed GF, Thoene JG, et al. Cysteamine therapy for children with nephropathic cystinosis. *N Engl J Med*. 1987;316(16):971-977. doi:10.1056/NEJM198704163161602 | Representative Image is accessible via reference |
| Gahl WA, Reed GF, Thoene JG, et al. Cysteamine therapy for children with nephropathic cystinosis. *N Engl J Med*. 1987;316(16):971-977. doi:10.1056/NEJM198704163161602 | Representative Image is accessible via reference |
| Gahl WA, Reed GF, Thoene JG, et al. Cysteamine therapy for children with nephropathic cystinosis. *N Engl J Med*. 1987;316(16):971-977. doi:10.1056/NEJM198704163161602 | Representative Image is accessible via reference |
| SLE image from participants | 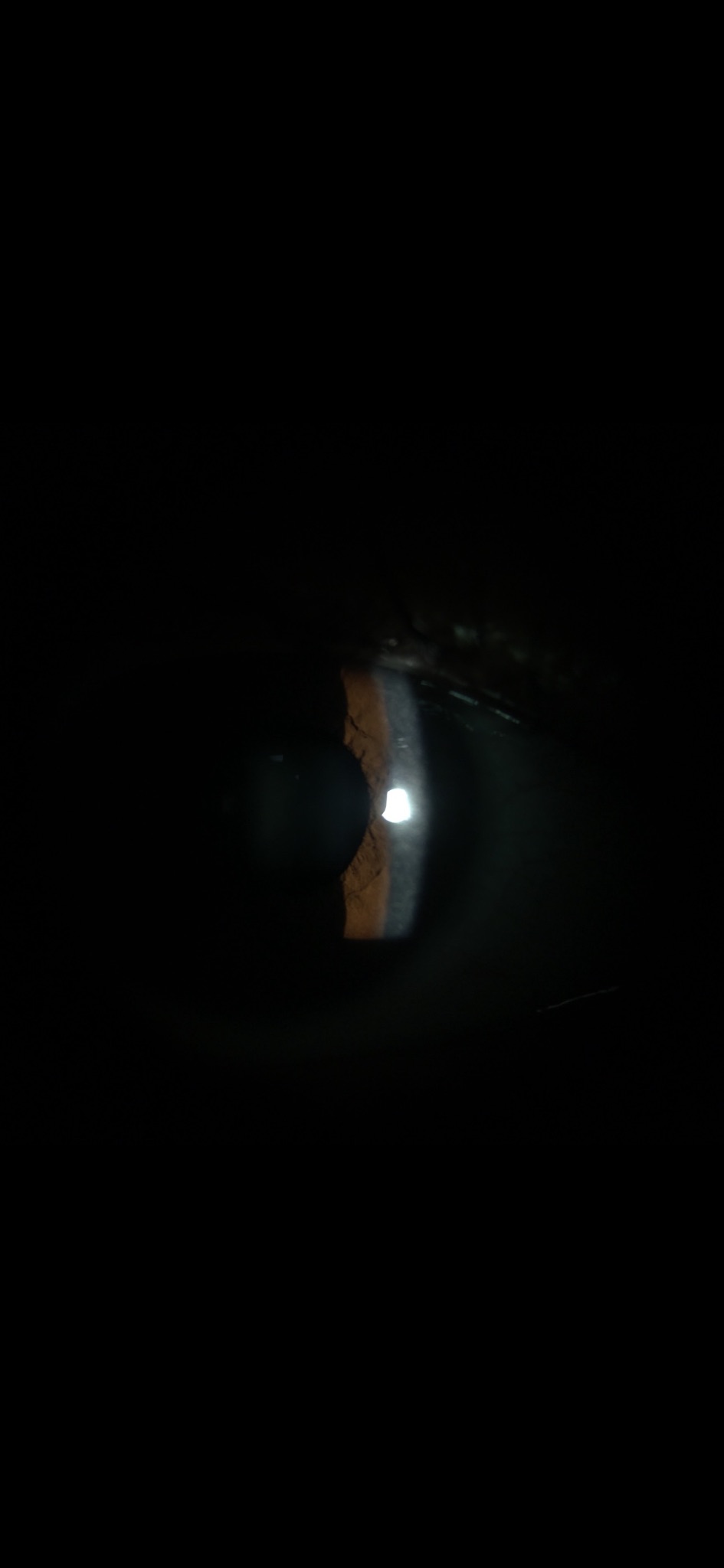 |
| Gahl WA, Reed GF, Thoene JG, et al. Cysteamine therapy for children with nephropathic cystinosis. *N Engl J Med*. 1987;316(16):971-977. doi:10.1056/NEJM198704163161602 | Representative Image is accessible via reference |
| Gahl WA, Reed GF, Thoene JG, et al. Cysteamine therapy for children with nephropathic cystinosis. *N Engl J Med*. 1987;316(16):971-977. doi:10.1056/NEJM198704163161602 | Representative Image is accessible via reference |
| Gahl WA, Reed GF, Thoene JG, et al. Cysteamine therapy for children with nephropathic cystinosis. *N Engl J Med*. 1987;316(16):971-977. doi:10.1056/NEJM198704163161602 | Representative Image is accessible via reference |
| SLE image from participants | 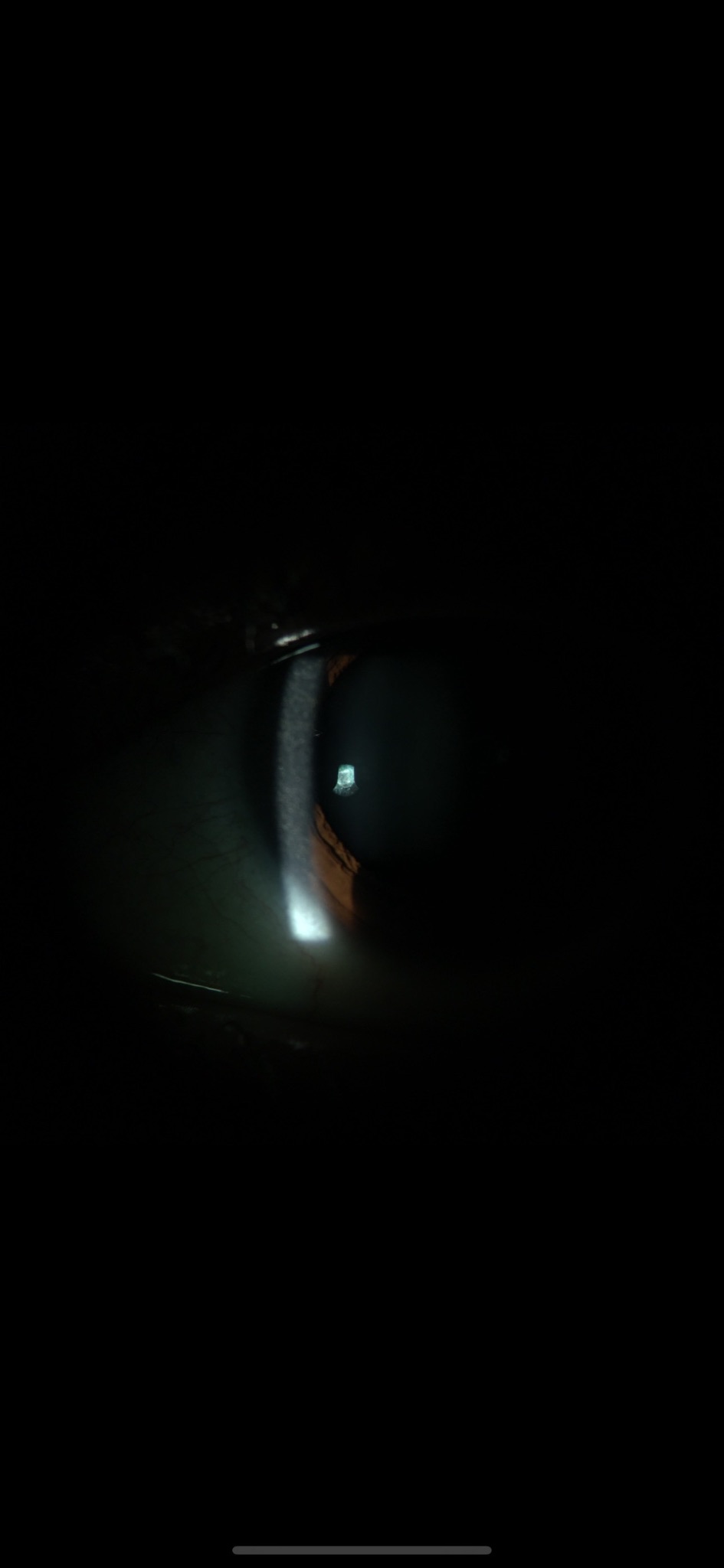 |
| SLE image from participants | 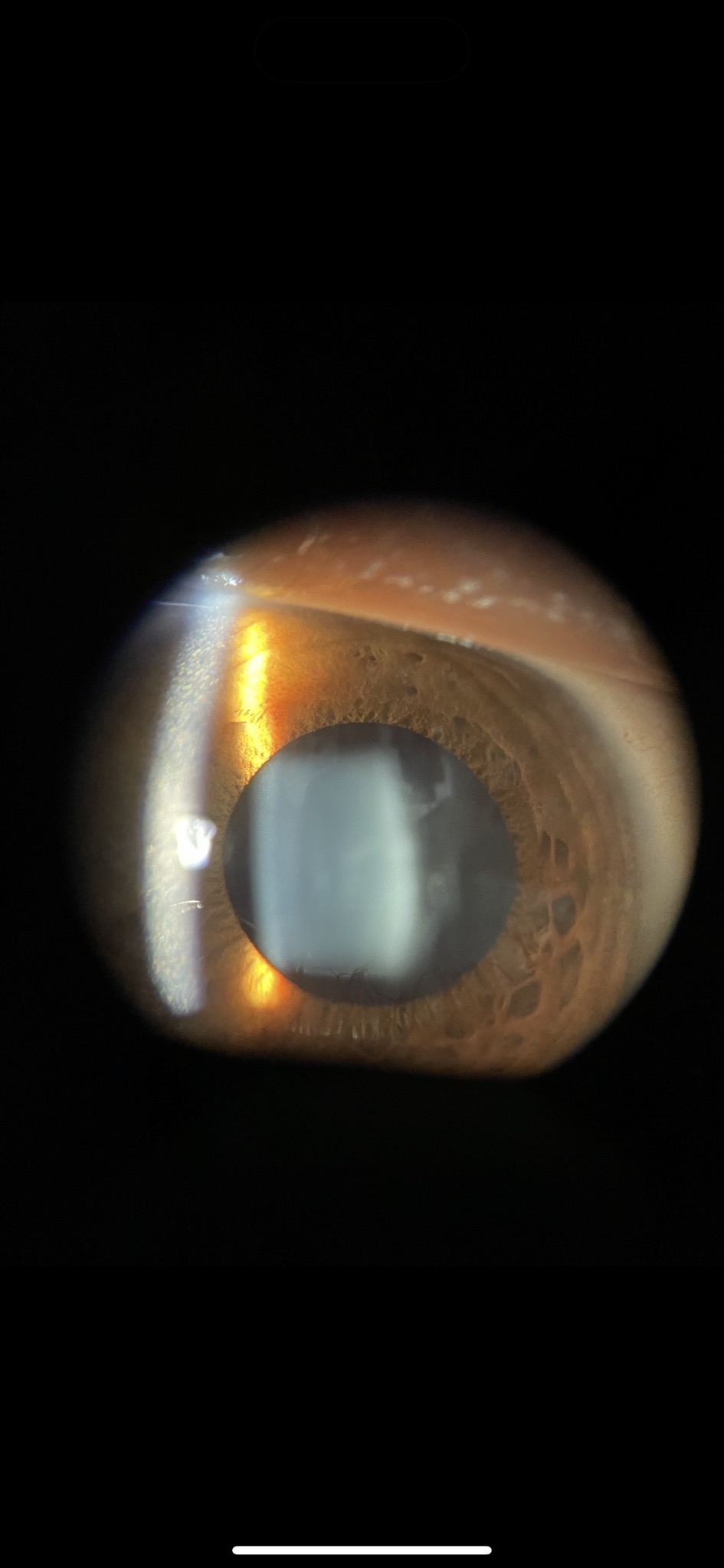 |
| Gahl WA, Reed GF, Thoene JG, et al. Cysteamine therapy for children with nephropathic cystinosis. *N Engl J Med*. 1987;316(16):971-977. doi:10.1056/NEJM198704163161602 | Representative Image is accessible via reference |
| Kaiser-Kupfer MI, Gazzo MA, Datiles MB, Caruso RC, Kuehl EM, Gahl WA. A randomized placebo-controlled trial of cysteamine eye drops in nephropathic cystinosis. *Arch Ophthalmol*. 1990;108(5):689-693. doi:10.1001/archopht.1990.01070070075038 | Representative Image is accessible via reference |
| Helmi HA, El Mansoury J, Al Hazzaa S, Al Zoba A, Dirar QS. Asymmetrical Ocular Manifestations of Nephropathic Cystinosis; A Case Report. *Am J Case Rep*. 2019;20:1308-1313. Published 2019 Sep 4. doi:10.12659/AJCR.916737 | Representative Image is accessible via reference |
| Gahl WA, Reed GF, Thoene JG, et al. Cysteamine therapy for children with nephropathic cystinosis. *N Engl J Med*. 1987;316(16):971-977. doi:10.1056/NEJM198704163161602 | Representative Image is accessible via reference |
| Al-Nabhani D, El-Naggari M, Al-Sinawi R, Chacko A, Ganesh A, Elnour I. Nephropathic cystinosis: First reported case in Oman. *Sultan Qaboos Univ Med J.* 2011;11(4):503-506. | Representative Image is accessible via reference |
| Gahl WA, Reed GF, Thoene JG, et al. Cysteamine therapy for children with nephropathic cystinosis. *N Engl J Med*. 1987;316(16):971-977. doi:10.1056/NEJM198704163161602 | Representative Image is accessible via reference |
| Kowalczyk M, Toro MD, Rejdak R, Załuska W, Gagliano C, Sikora P. Ophthalmic Evaluation of Diagnosed Cases of Eye Cystinosis: A Tertiary Care Center’s Experience. *Diagnostics*. 2020; 10(11):911. https://doi.org/10.3390/diagnostics10110911 | Representative Image is accessible via reference |
| Gahl WA, Reed GF, Thoene JG, et al. Cysteamine therapy for children with nephropathic cystinosis. *N Engl J Med*. 1987;316(16):971-977. doi:10.1056/NEJM198704163161602 | Representative Image is accessible via reference |
| Guignier B, Samet I, Bourcier T. Anterior segment spectral-domain optical coherence tomography findings in cystinosis. *Arch Ophthalmol*. 2012;130(8):1018. doi:10.1001/archophthalmol.2012.32 | Representative Image is accessible via reference |
| Bishop R. Ocular Complications of Infantile Nephropathic Cystinosis. *J Pediatr*. 2017;183S:S19-S21. doi:10.1016/j.jpeds.2016.12.055 | Representative Image is accessible via reference |
| ​​ ​​Helmi HA, El Mansoury J, Al Hazzaa S, Al Zoba A, Dirar QS. Asymmetrical Ocular Manifestations of Nephropathic Cystinosis; A Case Report. *Am J Case Rep*. 2019;20:1308-1313. Published 2019 Sep 4. doi:10.12659/AJCR.916737 | Representative Image is accessible via reference |
| Kowalczyk M, Toro MD, Rejdak R, Załuska W, Gagliano C, Sikora P. Ophthalmic Evaluation of Diagnosed Cases of Eye Cystinosis: A Tertiary Care Center’s Experience. *Diagnostics*. 2020; 10(11):911. https://doi.org/10.3390/diagnostics10110911 | Representative Image is accessible via reference |
| Pinxten AM, Hua MT, Simpson J, Hohenfellner K, Levtchenko E, Casteels I. Clinical Practice: A Proposed Standardized Ophthalmological Assessment for Patients with Cystinosis. *Ophthalmol Ther*. 2017;6(1):93-104. doi:10.1007/s40123-017-0089-3 | Representative Image is accessible via reference |
| SLE image from participants | 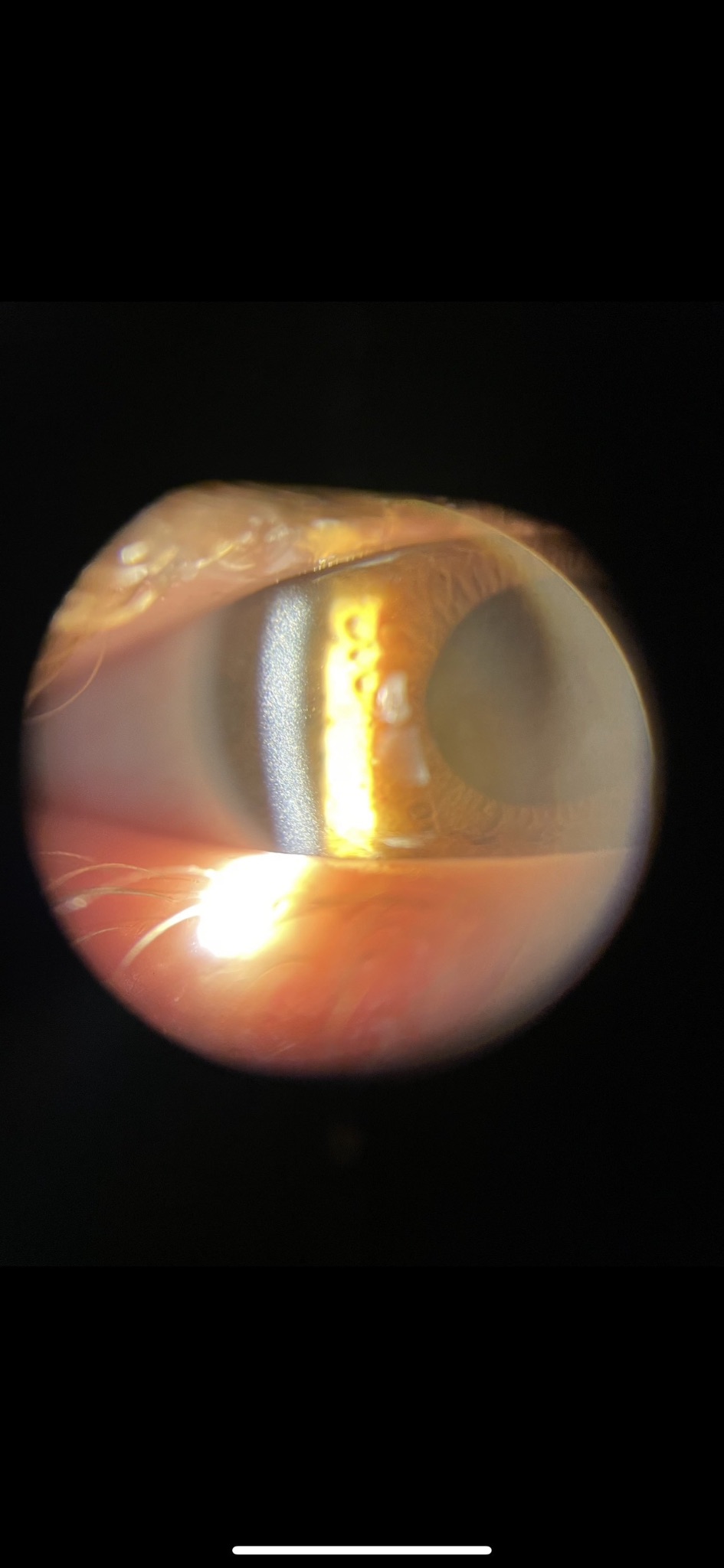 |
| Shams F, Livingstone I, Oladiwura D, Ramaesh K. Treatment of corneal cystine crystal accumulation in patients with cystinosis. *Clin Ophthalmol*. 2014;8:2077-2084. Published 2014 Oct 10. doi:10.2147/OPTH.S36626 | Representative Image is accessible via reference |
| Gahl WA, Reed GF, Thoene JG, et al. Cysteamine therapy for children with nephropathic cystinosis. *N Engl J Med*. 1987;316(16):971-977. doi:10.1056/NEJM198704163161602 | Representative Image is accessible via reference |
| Gahl WA, Reed GF, Thoene JG, et al. Cysteamine therapy for children with nephropathic cystinosis. *N Engl J Med*. 1987;316(16):971-977. doi:10.1056/NEJM198704163161602 | Representative Image is accessible via reference |
| Pinxten AM, Hua MT, Simpson J, Hohenfellner K, Levtchenko E, Casteels I. Clinical Practice: A Proposed Standardized Ophthalmological Assessment for Patients with Cystinosis. *Ophthalmol Ther*. 2017;6(1):93-104. doi:10.1007/s40123-017-0089-3 | Representative Image is accessible via reference |
| Shams F, Livingstone I, Oladiwura D, Ramaesh K. Treatment of corneal cystine crystal accumulation in patients with cystinosis. *Clin Ophthalmol*. 2014;8:2077-2084. Published 2014 Oct 10. doi:10.2147/OPTH.S36626 | Representative Image is accessible via reference |
| Helmi HA, El Mansoury J, Al Hazzaa S, Al Zoba A, Dirar QS. Asymmetrical Ocular Manifestations of Nephropathic Cystinosis; A Case Report. *Am J Case Rep*. 2019;20:1308-1313. Published 2019 Sep 4. doi:10.12659/AJCR.916737 | Representative Image is accessible via reference |
| American Academy of Ophthalmology. Cystinosis image. American Academy of Ophthalmology website. <https://www.aao.org/education/image/cystinosis> | Representative Image is accessible via reference |
| Kowalczyk M, Toro MD, Rejdak R, Załuska W, Gagliano C, Sikora P. Ophthalmic Evaluation of Diagnosed Cases of Eye Cystinosis: A Tertiary Care Center's Experience. *Diagnostics (Basel)*. 2020;10(11):911. Published 2020 Nov 7. doi:10.3390/diagnostics10110911 | Representative Image is accessible via reference |
| Shams F, Livingstone I, Oladiwura D, Ramaesh K. Treatment of corneal cystine crystal accumulation in patients with cystinosis. *Clin Ophthalmol*. 2014;8:2077-2084. Published 2014 Oct 10. doi:10.2147/OPTH.S36626 | Representative Image is accessible via reference |
| American Academy of Ophthalmology. Cystinosis image. American Academy of Ophthalmology website. <https://www.aao.org/education/image/cystinosis> | Representative Image is accessible via reference |
| SLE image from participants | 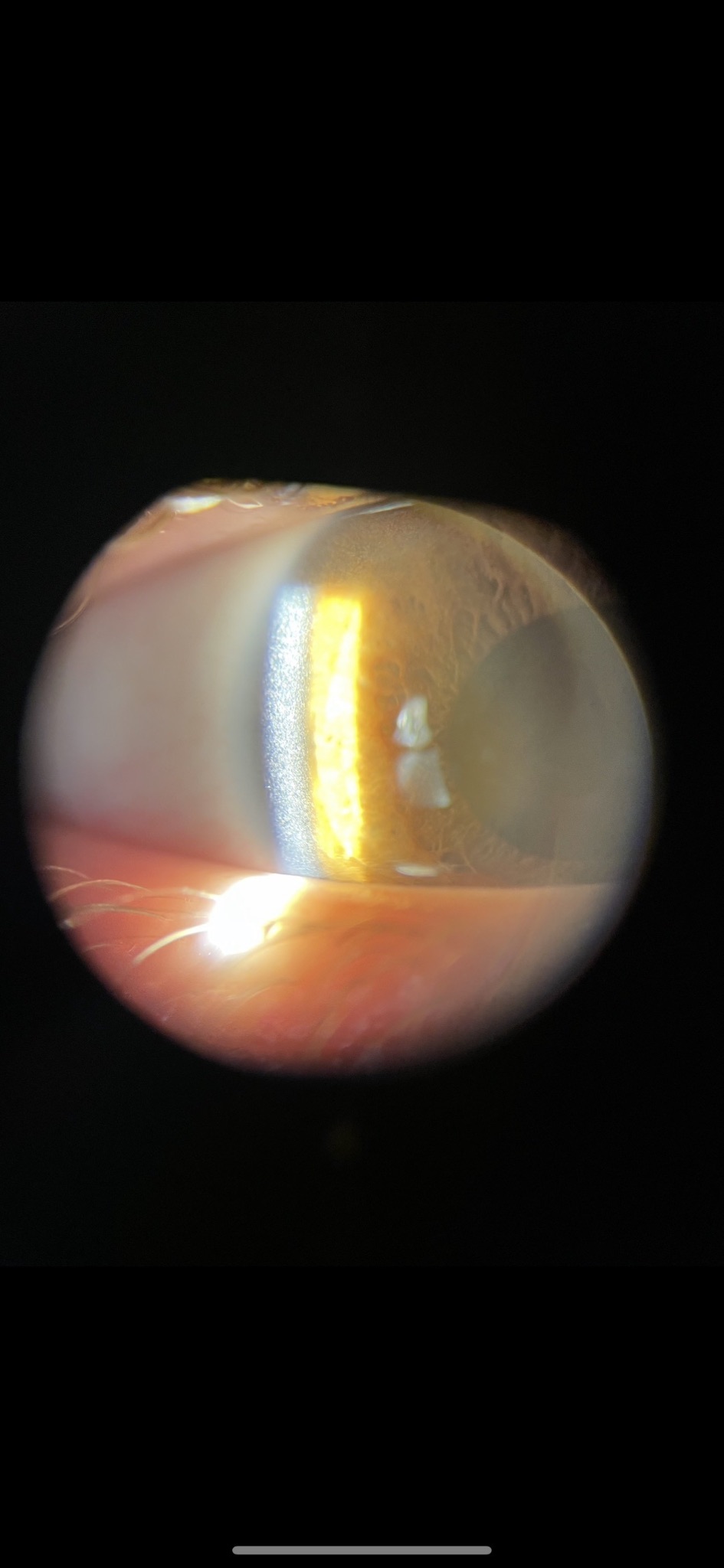 |
| Helmi HA, El Mansoury J, Al Hazzaa S, Al Zoba A, Dirar QS. Asymmetrical Ocular Manifestations of Nephropathic Cystinosis; A Case Report. *Am J Case Rep*. 2019;20:1308-1313. Published 2019 Sep 4. doi:10.12659/AJCR.916737 | Representative Image is accessible via reference |
| Helmi HA, El Mansoury J, Al Hazzaa S, Al Zoba A, Dirar QS. Asymmetrical Ocular Manifestations of Nephropathic Cystinosis; A Case Report. *Am J Case Rep*. 2019;20:1308-1313. Published 2019 Sep 4. doi:10.12659/AJCR.916737 | Representative Image is accessible via reference |
| American Academy of Ophthalmology. Cystinosis image. American Academy of Ophthalmology website. <https://www.aao.org/education/image/cystinosis> | Representative Image is accessible via reference |
| Helmi HA, El Mansoury J, Al Hazzaa S, Al Zoba A, Dirar QS. Asymmetrical Ocular Manifestations of Nephropathic Cystinosis; A Case Report. *Am J Case Rep*. 2019;20:1308-1313. Published 2019 Sep 4. doi:10.12659/AJCR.916737 | Representative Image is accessible via reference |
| Bishop R. Ocular Complications of Infantile Nephropathic Cystinosis. *J Pediatr*. 2017;183S:S19-S21. doi:10.1016/j.jpeds.2016.12.055 | Representative Image is accessible via reference |
| Gahl WA, Reed GF, Thoene JG, et al. Cysteamine therapy for children with nephropathic cystinosis. *N Engl J Med*. 1987;316(16):971-977. doi:10.1056/NEJM198704163161602 | Representative Image is accessible via reference |
| SLE image from participants | 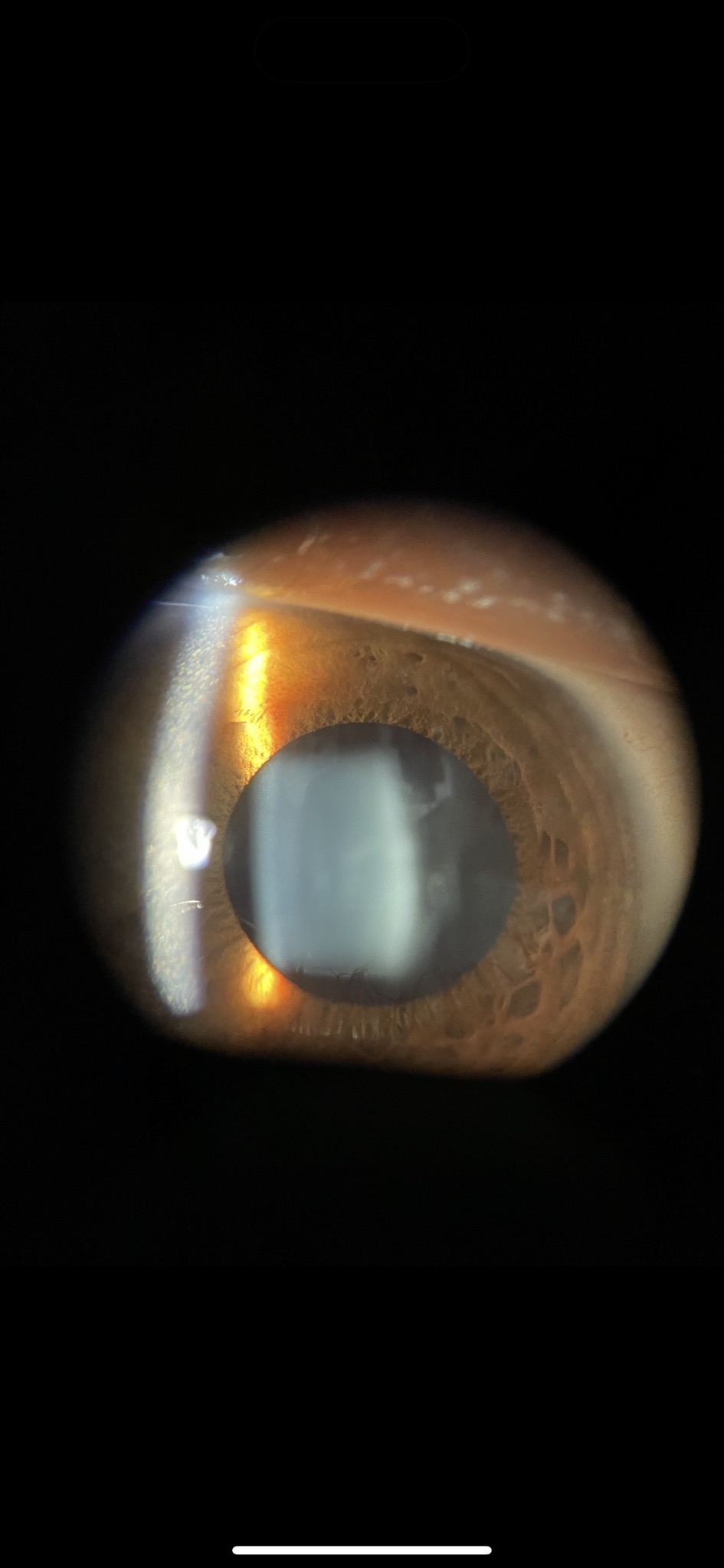 |
| Ophthalmic Atlas Images by EyeRounds.org, The University of Iowa. Cystinosis. <https://webeye.ophth.uiowa.edu/eyeforum/atlas/pages/cystinosis.htm#gsc>. tab=0. Accessed November 12, 2024. | Representative Image is accessible via reference |
| Bayram-Suverza M, Virgen-Batista MI, Vázquez-Lara Y. Importance of adherence to topical cysteamine in infantile ocular cystinosis: An illustrative case. *Indian J Ophthalmol*. 2022;70(7):2636-2638. doi:10.4103/ijo.IJO_2418_21 | Representative Image is accessible via reference |
| Kitnarong N, Osuwannaratana P, Kamchaisatian W, Namtongthai P, Metheetrairut A. Ocular manifestations in adolescent cystinosis: case report in Thailand. *J Med Assoc Thai*. 2005;88(4):521-526. | Representative Image is accessible via reference |
| Gahl WA, Reed GF, Thoene JG, et al. Cysteamine therapy for children with nephropathic cystinosis. *N Engl J Med*. 1987;316(16):971-977. doi:10.1056/NEJM198704163161602 | Representative Image is accessible via reference |
| Gahl WA, Reed GF, Thoene JG, et al. Cysteamine therapy for children with nephropathic cystinosis. *N Engl J Med*. 1987;316(16):971-977. doi:10.1056/NEJM198704163161602 | Representative Image is accessible via reference |
| Gahl WA, Reed GF, Thoene JG, et al. Cysteamine therapy for children with nephropathic cystinosis. *N Engl J Med*. 1987;316(16):971-977. doi:10.1056/NEJM198704163161602 | Representative Image is accessible via reference |
| Kitnarong N, Osuwannaratana P, Kamchaisatian W, Namtongthai P, Metheetrairut A. Ocular manifestations in adolescent cystinosis: case report in Thailand. *J Med Assoc Thai*. 2005;88(4):521-526. | Representative Image is accessible via reference |
| Gahl WA, Reed GF, Thoene JG, et al. Cysteamine therapy for children with nephropathic cystinosis. *N Engl J Med*. 1987;316(16):971-977. doi:10.1056/NEJM198704163161602 | Representative Image is accessible via reference |
| Gahl WA, Reed GF, Thoene JG, et al. Cysteamine therapy for children with nephropathic cystinosis. *N Engl J Med*. 1987;316(16):971-977. doi:10.1056/NEJM198704163161602 | Representative Image is accessible via reference |
| Ozdemir HB, Özmen MC, Aktas Z, Hasanreisoglu M. *In vivo* confocal microscopy and anterior segment optical coherence tomography follow-up of cysteamine treatment in corneal cystinosis. *Indian J Ophthalmol*. 2019;67(1):153-155. doi:10.4103/ijo.IJO_736_18 | Representative Image is accessible via reference |
| Bishop R. Ocular Complications of Infantile Nephropathic Cystinosis. *J Pediatr*. 2017;183S:S19-S21. doi:10.1016/j.jpeds.2016.12.055 | Representative Image is accessible via reference |
| Helmi HA, El Mansoury J, Al Hazzaa S, Al Zoba A, Dirar QS. Asymmetrical Ocular Manifestations of Nephropathic Cystinosis; A Case Report. *Am J Case Rep*. 2019;20:1308-1313. Published 2019 Sep 4. doi:10.12659/AJCR.916737 | Representative Image is accessible via reference |
| Ozdemir HB, Özmen MC, Aktas Z, Hasanreisoglu M. *In vivo* confocal microscopy and anterior segment optical coherence tomography follow-up of cysteamine treatment in corneal cystinosis. *Indian J Ophthalmol*. 2019;67(1):153-155. doi:10.4103/ijo.IJO_736_18 | Representative Image is accessible via reference |
| Bayram-Suverza M, Virgen-Batista MI, Vázquez-Lara Y. Importance of adherence to topical cysteamine in infantile ocular cystinosis: An illustrative case. *Indian J Ophthalmol*. 2022;70(7):2636-2638. doi:10.4103/ijo.IJO_2418_21 | Representative Image is accessible via reference |
| Bayram-Suverza M, Virgen-Batista MI, Vázquez-Lara Y. Importance of adherence to topical cysteamine in infantile ocular cystinosis: An illustrative case. *Indian J Ophthalmol*. 2022;70(7):2636-2638. doi:10.4103/ijo.IJO_2418_21 | Representative Image is accessible via reference |
| Gahl WA, Reed GF, Thoene JG, et al. Cysteamine therapy for children with nephropathic cystinosis. *N Engl J Med*. 1987;316(16):971-977. doi:10.1056/NEJM198704163161602 | Representative Image is accessible via reference |
| Gahl WA, Reed GF, Thoene JG, et al. Cysteamine therapy for children with nephropathic cystinosis. *N Engl J Med*. 1987;316(16):971-977. doi:10.1056/NEJM198704163161602 | Representative Image is accessible via reference |
| Gahl WA, Reed GF, Thoene JG, et al. Cysteamine therapy for children with nephropathic cystinosis. *N Engl J Med*. 1987;316(16):971-977. doi:10.1056/NEJM198704163161602 | Representative Image is accessible via reference |
| Gahl WA, Reed GF, Thoene JG, et al. Cysteamine therapy for children with nephropathic cystinosis. *N Engl J Med*. 1987;316(16):971-977. doi:10.1056/NEJM198704163161602 | Representative Image is accessible via reference |
| Bayram-Suverza M, Virgen-Batista MI, Vázquez-Lara Y. Importance of adherence to topical cysteamine in infantile ocular cystinosis: An illustrative case. *Indian J Ophthalmol*. 2022;70(7):2636-2638. doi:10.4103/ijo.IJO_2418_21 | Representative Image is accessible via reference |
| Gahl WA, Reed GF, Thoene JG, et al. Cysteamine therapy for children with nephropathic cystinosis. *N Engl J Med*. 1987;316(16):971-977. doi:10.1056/NEJM198704163161602 | Representative Image is accessible via reference |
| Gahl WA, Reed GF, Thoene JG, et al. Cysteamine therapy for children with nephropathic cystinosis. *N Engl J Med*. 1987;316(16):971-977. doi:10.1056/NEJM198704163161602 | Representative Image is accessible via reference |
| Gahl WA, Reed GF, Thoene JG, et al. Cysteamine therapy for children with nephropathic cystinosis. *N Engl J Med*. 1987;316(16):971-977. doi:10.1056/NEJM198704163161602 | Representative Image is accessible via reference |
| Gahl WA, Reed GF, Thoene JG, et al. Cysteamine therapy for children with nephropathic cystinosis. *N Engl J Med*. 1987;316(16):971-977. doi:10.1056/NEJM198704163161602 | Representative Image is accessible via reference |
| Gahl WA, Reed GF, Thoene JG, et al. Cysteamine therapy for children with nephropathic cystinosis. *N Engl J Med*. 1987;316(16):971-977. doi:10.1056/NEJM198704163161602 | Representative Image is accessible via reference |
| Gahl WA, Reed GF, Thoene JG, et al. Cysteamine therapy for children with nephropathic cystinosis. *N Engl J Med*. 1987;316(16):971-977. doi:10.1056/NEJM198704163161602 | Representative Image is accessible via reference |
| Gahl WA, Reed GF, Thoene JG, et al. Cysteamine therapy for children with nephropathic cystinosis. *N Engl J Med*. 1987;316(16):971-977. doi:10.1056/NEJM198704163161602 | Representative Image is accessible via reference |
| Gahl WA, Reed GF, Thoene JG, et al. Cysteamine therapy for children with nephropathic cystinosis. *N Engl J Med*. 1987;316(16):971-977. doi:10.1056/NEJM198704163161602 | Representative Image is accessible via reference |
| Gahl WA, Reed GF, Thoene JG, et al. Cysteamine therapy for children with nephropathic cystinosis. *N Engl J Med*. 1987;316(16):971-977. doi:10.1056/NEJM198704163161602 | Representative Image is accessible via reference |
| Gahl WA, Reed GF, Thoene JG, et al. Cysteamine therapy for children with nephropathic cystinosis. *N Engl J Med*. 1987;316(16):971-977. doi:10.1056/NEJM198704163161602 | Representative Image is accessible via reference |
| Gahl WA, Reed GF, Thoene JG, et al. Cysteamine therapy for children with nephropathic cystinosis. *N Engl J Med*. 1987;316(16):971-977. doi:10.1056/NEJM198704163161602 | Representative Image is accessible via reference |
| Gahl WA, Reed GF, Thoene JG, et al. Cysteamine therapy for children with nephropathic cystinosis. *N Engl J Med*. 1987;316(16):971-977. doi:10.1056/NEJM198704163161602 | Representative Image is accessible via reference |
| Gahl WA, Reed GF, Thoene JG, et al. Cysteamine therapy for children with nephropathic cystinosis. *N Engl J Med*. 1987;316(16):971-977. doi:10.1056/NEJM198704163161602 | Representative Image is accessible via reference |
| Gahl WA, Reed GF, Thoene JG, et al. Cysteamine therapy for children with nephropathic cystinosis. *N Engl J Med*. 1987;316(16):971-977. doi:10.1056/NEJM198704163161602 | Representative Image is accessible via reference |
| Gahl WA, Reed GF, Thoene JG, et al. Cysteamine therapy for children with nephropathic cystinosis. *N Engl J Med*. 1987;316(16):971-977. doi:10.1056/NEJM198704163161602 | Representative Image is accessible via reference |
| Gahl WA, Reed GF, Thoene JG, et al. Cysteamine therapy for children with nephropathic cystinosis. *N Engl J Med*. 1987;316(16):971-977. doi:10.1056/NEJM198704163161602 | Representative Image is accessible via reference |
| Gahl WA, Reed GF, Thoene JG, et al. Cysteamine therapy for children with nephropathic cystinosis. *N Engl J Med*. 1987;316(16):971-977. doi:10.1056/NEJM198704163161602 | Representative Image is accessible via reference |
| Gahl WA, Reed GF, Thoene JG, et al. Cysteamine therapy for children with nephropathic cystinosis. *N Engl J Med*. 1987;316(16):971-977. doi:10.1056/NEJM198704163161602 | Representative Image is accessible via reference |
| Gahl WA, Reed GF, Thoene JG, et al. Cysteamine therapy for children with nephropathic cystinosis. *N Engl J Med*. 1987;316(16):971-977. doi:10.1056/NEJM198704163161602 | Representative Image is accessible via reference |
| Gahl WA, Reed GF, Thoene JG, et al. Cysteamine therapy for children with nephropathic cystinosis. *N Engl J Med*. 1987;316(16):971-977. doi:10.1056/NEJM198704163161602 | Representative Image is accessible via reference |
| Gahl WA, Reed GF, Thoene JG, et al. Cysteamine therapy for children with nephropathic cystinosis. *N Engl J Med*. 1987;316(16):971-977. doi:10.1056/NEJM198704163161602 | Representative Image is accessible via reference |
| Gahl WA, Reed GF, Thoene JG, et al. Cysteamine therapy for children with nephropathic cystinosis. *N Engl J Med*. 1987;316(16):971-977. doi:10.1056/NEJM198704163161602 | Representative Image is accessible via reference |
| Gahl WA, Reed GF, Thoene JG, et al. Cysteamine therapy for children with nephropathic cystinosis. *N Engl J Med*. 1987;316(16):971-977. doi:10.1056/NEJM198704163161602 | Representative Image is accessible via reference |
| Gahl WA, Reed GF, Thoene JG, et al. Cysteamine therapy for children with nephropathic cystinosis. *N Engl J Med*. 1987;316(16):971-977. doi:10.1056/NEJM198704163161602 | Representative Image is accessible via reference |
| Gahl WA, Reed GF, Thoene JG, et al. Cysteamine therapy for children with nephropathic cystinosis. *N Engl J Med*. 1987;316(16):971-977. doi:10.1056/NEJM198704163161602 | Representative Image is accessible via reference |
| Gahl WA, Reed GF, Thoene JG, et al. Cysteamine therapy for children with nephropathic cystinosis. *N Engl J Med*. 1987;316(16):971-977. doi:10.1056/NEJM198704163161602 | Representative Image is accessible via reference |
| Gahl WA, Reed GF, Thoene JG, et al. Cysteamine therapy for children with nephropathic cystinosis. *N Engl J Med*. 1987;316(16):971-977. doi:10.1056/NEJM198704163161602 | Representative Image is accessible via reference |
| Gahl WA, Reed GF, Thoene JG, et al. Cysteamine therapy for children with nephropathic cystinosis. *N Engl J Med*. 1987;316(16):971-977. doi:10.1056/NEJM198704163161602 | Representative Image is accessible via reference |
| Gahl WA, Reed GF, Thoene JG, et al. Cysteamine therapy for children with nephropathic cystinosis. *N Engl J Med*. 1987;316(16):971-977. doi:10.1056/NEJM198704163161602 | Representative Image is accessible via reference |
| Gahl WA, Reed GF, Thoene JG, et al. Cysteamine therapy for children with nephropathic cystinosis. *N Engl J Med*. 1987;316(16):971-977. doi:10.1056/NEJM198704163161602 | Representative Image is accessible via reference |
| Gahl WA, Reed GF, Thoene JG, et al. Cysteamine therapy for children with nephropathic cystinosis. *N Engl J Med*. 1987;316(16):971-977. doi:10.1056/NEJM198704163161602 | Representative Image is accessible via reference |

**Supplementary Figure 1:** Comprehensive collection of slit lamp photographs selected from open-access articles available on PubMed. (Note: Images originally obtained from open-access literature have been removed to comply with copyright guidelines; the representative lit lamp photographs remain publicly accessible through the cited references.)
